# A Rare and Potentially Fatal Etiology of Hypercalcemia in an Infant

**DOI:** 10.1155/2019/4270852

**Published:** 2019-08-05

**Authors:** Ambreen Sonawalla, Vildan Tas, Manish Raisingani, Emir Tas

**Affiliations:** ^1^Pediatric Residency Program, University of Arkansas for Medical Sciences, Little Rock, AR, USA; ^2^Department of Pediatrics, University of Arkansas for Medical Sciences, Little Rock, AR, USA; ^3^Department of Pediatrics, Division of Endocrinology and Diabetes, University of Arkansas for Medical Sciences, Little Rock, AR, USA

## Abstract

Hypercalcemia is an uncommon finding in children. Hypercalcemia has various etiologies including parathyroid dependent and independent mechanisms. Increased activity of the 1-alpha-hydroxylase enzyme in granulomatous diseases is a well-defined but an extremely rare cause of hypercalcemia in pediatric patients, particularly in infants. We describe the case of an infant who presented with failure to thrive, hepatosplenomegaly, and hypercalcemia who was initially treated with steroids but was later diagnosed with disseminated histoplasmosis in the absence of an underlying immunodeficiency. Extra caution should be used before considering steroids for the treatment of hypercalcemia and, whenever possible, steroids should not be initiated until a definite etiology is identified.

## 1. Introduction

Serum calcium level is tightly regulated as calcium is important for numerous cellular functions including cell membrane integrity, muscle contraction, and neuronal excitability [1]. Calcium regulation involves a very-well orchestrated interplay between parathyroid hormone, 1,25-dihydroxy vitamin D, phosphorus, and multiple organ systems including the gut, kidneys, skin, and liver.

Hypercalcemia is an infrequently encountered biochemical abnormality in the pediatric age group [2]. The most common causes, unlike in adults, are parathyroid hormone independent processes. Among them, granulomatous diseases, such as fungal infections, are an uncommon but recognized cause of hypercalcemia [3]. Granulomatous diseases lead to increased activity of the 1alpha-hydroxylase enzyme which converts 25-hydroxy vitamin D to 1,25-dihydroxy vitamin D, the biologically active form.

Hypercalcemia of any cause is initially treated symptomatically, with the ultimate treatment decided by the underlying cause. Steroids have typically been used in the treatment of hypercalcemia when it is secondary to excess vitamin D ingestion or increased 1alpha-hydroxylase activity such as in granulomatous disorders [4].

## 2. Case Presentation

A 7-month-old ex-30-week preterm female infant presented to the emergency department of our hospital with a two-month history of worsening intermittent vomiting and failure to thrive despite nutritional optimization and trial of different infant formulas. Past medical history was notable for prolonged NICU stay mainly due to delays in oral feeding. She had no pulmonary, cardiac, or intestinal complications of prematurity. She did not require any surgical procedure or hospital admission following NICU discharge at around 6 weeks of age. History is also negative for recent fevers or recurrent infections.

On presentation, she was in no acute distress and had normal vital signs. Weight and height were both below the first percentile. Physical examination was significant for the presence of hepatosplenomegaly. Laboratory workup was notable for elevated transaminases and significant hypercalcemia (4.47 mmol/L; Normal Range: 2.12-2.74mmol/L). Baseline phosphorus level was normal and the parathyroid hormone level was appropriately suppressed as seen in Table 1. She was admitted to hospital for management and further workup.

Aggressive intravascular fluid resuscitation with normal saline only partially improved serum calcium levels. Furosemide and calcitonin were used in succession, but they also failed to have a noticeable impact on serum calcium levels. Two doses of pamidronate, 0.5 mg/kg each two days apart, were eventually successful in restoring normal calcium levels. She also switched to low calcium infant formula, Calcilo- XD.

An extensive workup for viral and fungal etiologies was negative, as was the evaluation for metabolic, genetic, and oncologic causes of hypercalcemia. The skeletal survey did not show any lytic lesions; CT scan of the chest, abdomen, and pelvis was negative for the presence of lymphadenopathy or pulmonary lesions. Hepatosplenomegaly was confirmed with a CT scan; however, the underlying pathology was not revealed until a liver biopsy was performed for persistently elevated liver enzymes and massive hepatomegaly. Liver biopsy showed lobular histiocytic infiltrate with well-formed granulomas, hemophagocytosis, and increased portal/periportal and pericellular fibrosis with bridging indicating chronicity, with no further evidence to indicate an underlying etiology of the granulomas.

She was discharged home after 3 weeks of hospitalization once adequate weight gain and normal and stable calcium levels were achieved. Liver enzymes were improved but remained elevated. She had required readmission after one month for the recurrence of vomiting and poor weight. She had no fevers. Workup on this admission showed leukopenia and return of hypercalcemia (Table 1). Chronic granulomatous disease, immunodeficiency, tuberculosis, and hemophagocytic lymphohistiocytosis (HLH) were considered in the differential; however, screening was negative. HLH genotyping showed only a single allele mutation on UNC13 gene, but this variant did not explain her findings. Despite the lack of a standard definition of infantile sarcoidosis, this disease was considered due to elevated levels of angiotensin-converting enzyme (ACE) at 108 U/L (18-90). In the light of negative results for a possible underlying immunodeficiency and malignancy, she was placed on prednisolone 1 mg/kg/day for hypercalcemia. She remained afebrile and was discharged home in stable condition with prednisolone.

After 5 weeks of prednisolone treatment, she presented with daily emesis and low-grade fevers. Complete blood count showed pancytopenia, peripheral smear showed fungal elements. She was admitted to the intensive care unit for disseminated fungal infection. Urine Histoplasma antigen was found to be positive. Systemic antifungal treatment was started. Of note, serum calcium and liver enzyme levels were normal during the third admission (Table 1). Because of disseminated fungal infection, prednisolone was discontinued and a hydrocortisone taper was initiated. She had an excellent response to antifungal treatment. Hepatosplenomegaly resolved and all other serum markers have improved. She remained on systemic antifungal treatment for 9 months. She made full-recovery and caught up with growth and development.

## 3. Discussion

Hypercalcemia results when there is increased bone resorption, excessive intestinal absorption, or decreased renal excretion of calcium, or a combination of these processes. It may be asymptomatic or present with hypotonia, poor feeding, vomiting, failure to thrive (FTT), constipation, polyuria, dehydration, seizures, or, even psychiatric symptoms depending on the age of the patient [5]. Establishing the etiology requires concomitant evaluation of serum total and ionized calcium, phosphorus, magnesium, intact parathyroid hormone, 25-hydroxy vitamin D (25(OH)D_3_), and 1,25-dihydroxy vitamin D (1,25(OH)_2_D_3_) concentrations, as well as renal function and urinary calcium excretion [4]. Unlike in adults, hypercalcemia in children is most commonly secondary to parathyroid hormone independent processes. These include hypervitaminosis D and A, medications, malignancies, granulomatous disorders, endocrinopathies, renal tubular disorders, chronic inflammatory disorders, and infections [5].

Hypervitaminosis D may be from elevated levels of 25(OH)D_3_ concentrations, which is usually due to excess ingestion of vitamin D, or from elevated levels of 1,25(OH)_2_D_3_ which may be from malignancy, granulomatous diseases, subcutaneous fat necrosis of the newborn, or idiopathic infantile hypercalcemia [5].

Granulomatous diseases, including fungal infections, are a rare but well-established cause of hypercalcemia [6]. The mechanism is extra-renal production of 1,25(OH)_2_D_3_ by 1alpha-hydroxylase in activated macrophages within the granulomas, best described in sarcoidosis [7]. The elevated level of the active vitamin D metabolite causes hypercalcemia by a combination of increased intestinal absorption and reduced bone resorption of calcium, as well as increased renal tubular reabsorption. These macrophages are not responsive to negative feedback from elevated levels of 1,25(OH)_2_D_3_ but respond to steroids.

Initial management of hypercalcemia depends on the presence of symptoms and degree of elevation of serum calcium, but ultimate treatment depends on the underlying cause. Symptomatic patients are treated with hydration and furosemide. If hypercalcemia persists, calcitonin and bisphosphonates may be used. The latter is effective especially when the primary cause of the hypercalcemia is increased mobilization of bone calcium. Our patient's severe, symptomatic hypercalcemia (Table 1) did not respond to any of the first line treatments. She had a temporary response to IV bisphosphonates but returned with hypercalcemia after only 4 weeks of the infusion therapy as the bisphosphonate did not target the underlying etiology. The role of steroids in the treatment of hypercalcemia in granulomatous diseases is well-established [3, 5]. Steroids exert their action by suppressing the excess 1alpha-hydroxylase activity and decreasing extra-renal production of 1,25(OH)_2_D_3_ [8]. Prednisone or prednisolone at a dose of 1 mg/kg/day divided into two doses is the recommended treatment regimen [4]. Our patient's calcium level remained normal and stable following initiation of the steroid treatment.

Histoplasmosis, a granulomatous fungal infection, is the most common endemic fungus in the United States. Most patients remain asymptomatic and have a self-limited illness [9]. Fever, respiratory symptoms, lymphadenopathy, splenomegaly, and poor weight gain are among the most common signs and symptoms, but hypercalcemia is a rare manifestation. None of the 40 infants described by Odio et al. had hypercalcemia as a finding of disseminated histoplasmosis [10]. Surprisingly, our patient had an atypical presentation for fungal infection and did not present with any of the classic symptoms.

The literature is scarce on hypercalcemia in children and adults with histoplasmosis. Steele et al. [11] described a 10-month-old infant with disseminated histoplasmosis who presented with FTT, vomiting, hepatosplenomegaly, and hypercalcemia. Hypercalcemia in this infant was successfully managed with dietary calcium restriction alone; however, it is not clear whether the timing of antifungal treatment in the course of the fungal infection had prevented worsening hypercalcemia. Surprisingly, the infant had to be maintained on a low calcium diet even at 3-year follow up. This is in contrast to our patient who, though was initially placed on a low calcium and vitamin D formula, did not continue to require it when the underlying cause of the hypercalcemia, the fungal infection, had been controlled. Two adult cases with histoplasmosis related hypercalcemia were initially diagnosed with sarcoidosis based on the presenting clinical and laboratory data. One of the patients was found to have histoplasmosis soon after steroid treatment was begun for presumed sarcoidosis [12]. Hypercalcemia in this case resolved with the treatment of the fungal infection. The patient described by Walker et al. [13], however, presented with prolonged malaise, fever, nausea, vomiting, and weight loss. He was readmitted to the hospital after initiation of steroids, with recurrent fever and died from widespread organ disease and cardiac arrest. A postmortem examination found evidence of disseminated histoplasmosis.

Our patient was also initially placed on steroid treatment due to presumed sarcoidosis. Although there are no well-established diagnostic criteria for infantile sarcoidosis, the constellation of her signs and symptoms along with laboratory data including elevated angiotensin-converting enzyme activity in the absence of other explanation for hypercalcemia was the reason of this decision. The correct diagnosis was reached when she presented with pancytopenia, a few weeks into prednisone treatment.

## 4. Conclusion

Hypercalcemia is an uncommon biochemical abnormality in the pediatric age group. Disseminated fungal infections such as histoplasmosis are also uncommon in an otherwise healthy infant, and hypercalcemia is a rare presenting symptom in such patients. However, even in the absence of classic signs of fungal infections, as was the case in our patient, it is important to consider fungal causes in hypercalcemia with granulomas and evidence of hypervitaminosis D.

Steroids are a well-established treatment for hypercalcemia in granulomatous diseases. Although steroids may resolve several of the overlapping symptoms, they can also unmask the fungal infection and have potentially lethal consequences. It is therefore important to exclude infectious granulomatous etiologies prior to initiating steroid therapy for hypercalcemia.

## Figures and Tables

**Table 1:** Calcium metabolism markers at each of the hospital admissions.

	Initial presentation	Second admission	Third admission
	(Baseline)	(+7 weeks)	(+15 weeks)
*Total calcium* (RR: 2.12-2.74 mmol/L)	4.47	3.37	2.47

*Ionized calcium* (RR: 1.1-1.3 mmol/L)	2.43	1.83	1.35

*25 hydroxy vit D* (RR: 20-200 nmol/L)	153		50.2

*1,25 dihydroxy vit D* (RR: 49.7-197.9 pmol/L)	364		92.9

*Parathyroid hormone* (RR: 12-65 ng/L)	5.3		

*Phosphorus* (RR: 1.39-2.39 mmol/L)	1.55		1.1

*Albumin* (RR: 3.5 – 4.5 g/L)	4.4	4.1	2.4

*Urine Ca:Cr ratio*	1.85		
